# Concussion history and virtual reality metrics predict core or lower extremity injury occurrence among high school athletes

**DOI:** 10.3389/fspor.2024.1374772

**Published:** 2024-03-27

**Authors:** Gary B. Wilkerson, Kimberly R. Wynn, Paige W. Dill, Shellie Acocello, Lynette M. Carlson, Jennifer Hogg

**Affiliations:** ^1^ Department of Health and Human Performance, University of Tennessee at Chattanooga, Chattanooga, TN, United States; ^2^ Department of Intercollegiate Athletics, Mercer University, Macon, GA, United States; ^3^ Sports Medicine Outreach Program, Optim Health System, Mount Vernon, GA, United States

**Keywords:** mild traumatic brain injury, injury risk, injury prevention, clinical assessment, choice reaction time

## Abstract

**Introduction:**

A history of concussion is recognized as a risk factor for musculoskeletal injury, which is likely associated with physiological effects that warrant better understanding. This study aimed to assess the potential of measurements obtained from an immersive virtual reality (VR) test to identify a subtle perceptual–motor impairment that may be prospectively associated with the occurrence of a core or lower extremity sprain or strain.

**Methods:**

A cohort of 68 high school athletes (41 female soccer players and 27 male football players) provided survey responses and completed an immersive VR test several days prior to the initiation of preseason practice sessions. Measurements of eye, neck, arm, and whole-body displacements were obtained during 40 successive lunging/reaching responses to visual stimuli moving horizontally across the VR headset display. Injury occurrences were electronically documented from the initial preseason practice session to the final game of the season.

**Results:**

A statistically significant and intrinsically credible two-factor prediction model for core or lower extremity injury occurrence included an interaction between female sex and a self-reported history of two or more concussions, along with slow response time (RT) for arm reach (OR = 4.67; 95% CI, 1.51–14.43). Follow-up analyses identified sex-specific cut points for arm reach RT associated with elevated injury risk, which were ≥1.385 s for females and ≥1.257 s for males.

**Discussion:**

High school female soccer players who have sustained more than one concussion appear to be highly vulnerable to core or lower extremity sprain or strain, with the risk of injury compounded by a slow arm reach RT. Male football players as a group demonstrated significantly faster arm reach RT than that of female soccer players, but slow perceptual–motor RT for arm reach was also identified as a potentially important injury risk factor for male players. Immersive VR appears to provide precise measurements of behavioral performance characteristics that depend on brain processing efficiency. Given that the speed, accuracy, and consistency of perceptual–motor responses may be modifiable, future research should explore the potential benefits of VR training for reducing the risk of sport-related injuries.

## Introduction

A history of concussion (HxC) has been recognized as a risk factor for subsequent musculoskeletal injury (1–5) and elevated susceptibility for another concussion and cumulative neural impairment (6–13). Advanced neuroimaging methods have demonstrated physiological effects that persist for months or years beyond clinical recovery (6, 7, 13–15). However, they are not feasible for routine assessments (16), and standard clinical tests lack sensitivity to detect subtle impairments (8, 16–20). Although the exact mechanism responsible for the elevation of post-concussion injury risk is unknown (21, 22), neuromechanical responsiveness to changing environmental conditions may be delayed by deficits in visual–spatial attention and cognitive–motor integration (i.e., perceptual–motor processing) (12, 23–25). Because the self-reported resolution of concussion symptoms is currently the primary criterion for return to sport participation, there exists an urgent need for a clinical test that can identify individual athletes who possess an elevated risk for further injury (12, 14, 15).

Clinical neurocognitive tests typically measure responses to various types of stimuli through simple motor responses, such as button presses or mouse clicks. Assessments of whole-body movement have not typically imposed a simultaneous cognitive demand, but dual-task protocols that require the engagement of both cognitive and motor processes have been increasingly advocated for post-concussion assessment (17, 21, 25). Postural balance or walking gait are typically measured in isolation, followed by the addition of a secondary cognitive demand that permits quantification of the incremental dual-task cost imposed by activities such as spelling words backward, reciting months in reversed chronological order, and serial subtractions (26). Clinical testing that effectively challenges cognitive control of goal-directed behavior, including visual–spatial attention, perceptual detection/identification, conflict resolution, restraint of impulsivity, working memory, and motor programming/control subcomponents, seems most likely to detect a subtle deficiency in the integration of visual, cognitive, and motor processes (12, 21, 23–25, 27–30).

A clinical test of integrated neural processing should simulate the demands imposed during sport participation to the greatest extent possible (25). Virtual reality (VR) testing administered with a head-mounted display offers a means to create a sense of immersion that can approximate real-world responses to dynamic visual stimuli (31). An external focus on visual stimuli that direct responses may facilitate a naturalistic self-organization of the musculoskeletal system (32), which may enhance the detection of perceptual–motor disintegration (33). There is some evidence that VR training may reduce sport-related injury risk (34), but relatively few studies have investigated the potential value of VR for concussion management (31, 33). Specific features of VR test design and the measurements chosen to quantify performance may be critical factors for the identification of subtle impairments (35).

Concussion causes diffuse axon injury that prolongs neural processing of sensory information that is manifested as slow response time (RT) averaged over multiple trials (RT-Avg) and suboptimal response accuracy. Prioritization of either speed or accuracy presents a trade-off that can be resolved through the calculation of a composite measure representing both aspects of stimulus responses, such as rate correct score (RCS: number of correct responses per second of cumulative RT for all trials) (36). In addition to the generation of fast and accurate responses, efficient brain processing produces relatively consistent response times over multiple trials (37, 38). Low intraindividual variability (IIV) among stimulus responses (e.g., standard deviation or coefficient of variation) has been demonstrated to provide meaningful information about brain processing efficiency that is not derived from a measure of central tendency (e.g., RT-Avg) (20, 28, 29, 35, 39–41).

Repeated concussions have been shown to increase the severity of both cognitive and emotional symptoms, which could conceivably result from a common pathophysiological mechanism (8). Lack of access to complete medical records typically makes survey acquisition of self-reported HxC and self-ratings of various aspects of physical, emotional, and sleep-related well-being the only feasible means to document the existence of potential contributors to elevated injury risk. Several previous studies have confirmed an association between HxC and subsequent musculoskeletal injury among adolescent athletes (22, 25, 42, 43), but only one study has been focused on the identification of perceptual–motor deficiencies that may elevate their injury susceptibility (23). Thus, the purpose of this prospective cohort study was to identify factors derived from survey responses and a VR test of perceptual–motor performance and possible interactions between factors that most strongly predict the subsequent occurrence of a core or lower extremity injury (CLEI) among male and female high school athletes.

## Methods

### Participants

A cohort of 68 athletes from two private high schools comprised 41 female soccer players (14.9 ± 0.9 years, 165.0 ± 6.3 cm, 58.6 ± 6.6 kg) and 27 male football players (15.8 ± 1.5 years, 178.2 ± 7.8 cm, 80.1 ± 18.9 kg), who had parent/guardian permission to provide survey responses and to perform an immersive VR test several days prior to the initiation of preseason practice sessions in mid-July of 2022. All study procedures were approved by the Institutional Review Board of the University of Tennessee at Chattanooga. Exclusionary criteria were lack of parent/guardian consent documentation, lack of athlete assent, or an injury-related limitation in the ability to perform simultaneous reaching and lunging movements.

### Procedures

The Global Well-Being Index (GWBI) was electronically administered (REDCap, Vanderbilt University, Nashville, TN, USA) (44) to quantify physical, behavioral, and emotional status on a 0–100 scale. The problem categories and specific conditions included in the GWBI survey were derived from prior analyses that identified those most frequently reported by athletes for the 10-item sport fitness index and the 82-item overall wellness index surveys (45–49). The GWBI assigns point values between 0 and 10 for each of the five problem categories that each include three specific conditions: general pain or discomfort (headaches/pressure in head, neck pain, non-specific body discomfort), sleep-related problems (trouble falling asleep, sleeping less, fatigue/drowsiness), mood-related problems (nervousness/anxiety, sadness/depression, irritability/stress), musculoskeletal problems (aching discomfort, joint stiffness, muscle spasms/tightness), and high-intensity performance limitations (running speed limitation, explosive power limitation, endurance limitation). Responses to a set of three follow-up questions rated frequency of occurrence (1–3 points), most recent occurrence (1–4 points), and severity over the past couple of years (1–3 points) for the worst condition identified within a category. A high raw score (50 points maximum) represents suboptimal status, whereas the 0–100 GWBI is derived from the multiplication of the raw score by 2 and subtraction from 100 (i.e., high = good).

**Figure 1:**
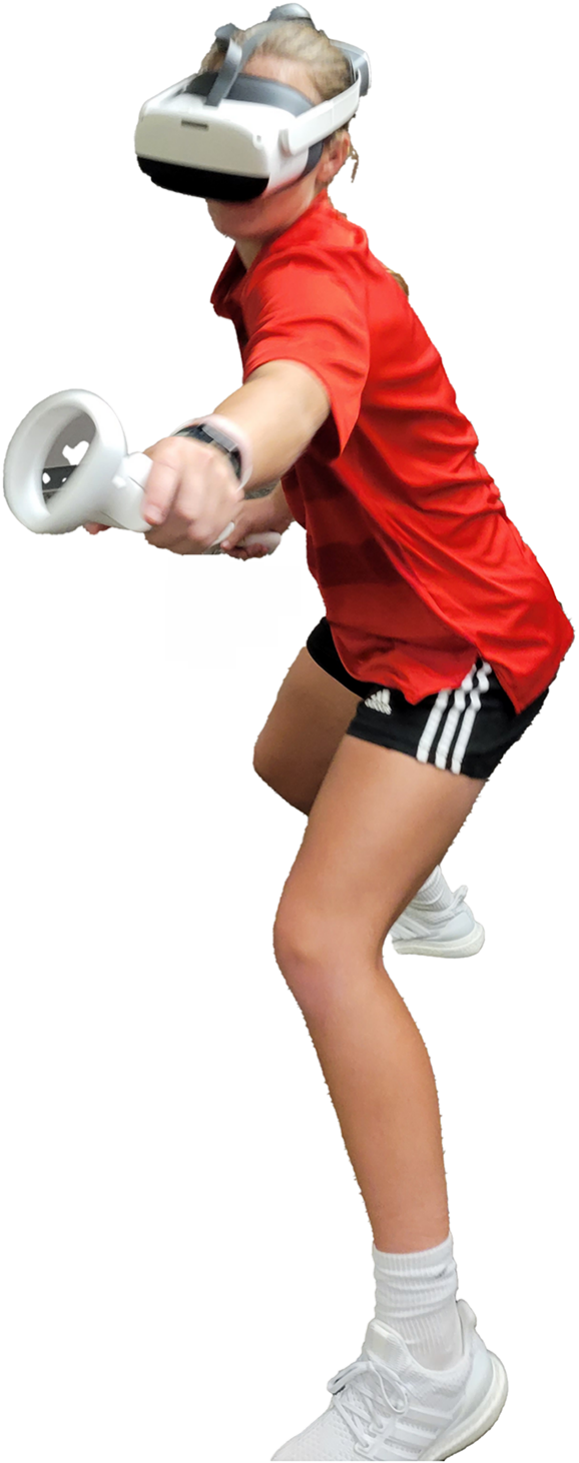
Reaching/lunging movement to contact virtual target in response to a visual stimulus moving horizontally across the virtual reality (VR) headset display.

The investigational VR test, not approved by the FDA for any purpose, required simultaneous neck rotation, arm reaching, and whole-body lunging movements in left or right directions (Figure 1) in response to the characteristics of visual stimuli moving horizontally across the black background of a head-mounted display (PICO Neo3 Pro Eye, PICO Immersive, Ltd., Mountain View, CA, USA). The stimuli initially appeared at either the center of the visual display or its left or right peripheral margins and moved in either a left-to-right or right-to-left direction. If the visual stimulus was a filled white circle, the correct directional response corresponded to the direction of its movement (i.e., congruent stimulus–response). If the visual stimulus was a white ring, the correct directional response was opposite to that of the movement of the ring (i.e., incongruent stimulus–response). To complete a trial, a hand controller needed to make virtual contact with a peripherally located response target (a green sphere that was not visible without neck rotation and a lunging/reaching movement). The distance to the response target was 30% beyond maximum arm reach, which was derived from a pretest measurement of the horizontal wingspan of the athlete. Both an auditory tone and vibration of the hand controller confirmed response target contact. The 40-trial immersive VR test presented eight different combinations of stimulus initial position, stimulus type, and movement direction. The validity of the test for discrimination between individuals with and without a lifetime history of concussion has been established (47) and documentation of moderate to excellent test–retest reliability for the various measurements derived from the test (50).

Perceptual latency was defined as the time that elapsed from stimulus appearance to 6° of neck rotation, 10 cm of arm (i.e., hand controller) movement, or 10 cm of step (i.e., whole-body lunge) displacement, with movement time representing the period from movement onset to response completion. The RT was defined as the time that elapsed from stimulus appearance to maximum neck, arm, or step displacement (i.e., RT = perceptual latency + movement time). In addition to the 40-trial average of perceptual latency (PL-Avg) and response time (RT-Avg) for neck, arm, and step movements, the 40-trial IIV (i.e., standard deviation) of perceptual latency (PL-IIV) and response time (RT-IIV) was calculated for the neck, arm, and side-step movements of each athlete. To acquire an integrated representation of speed-accuracy trade-off, the RCS was calculated as number of correct arm (i.e., hand controller) responses divided by the sum of either perceptual latency (RCS-PL) or response time (RCS-RT) for all trials. Thus, RCS-PL reflects the speed and accuracy of primarily perceptual processes, whereas RCS-RT incorporates the combined durations of the perceptual and movement phases of stimulus–response.

All injury occurrences during practice sessions or games were electronically documented by athletic trainers. The outcome of interest was the occurrence of a core (i.e., abdomen, low back, or pelvis) or lower extremity injury (CLEI), which was further defined as any sprain or strain that interrupted participation in a practice session or game and that received some type of treatment, regardless of whether participation was restricted on a subsequent date.

### Statistical analysis

To assess the potential for prediction of CLEI occurrences, a receiver operating characteristic (ROC) analysis was performed for each continuous measure. Area under the curve (AUC) values were interpreted as potentially useful in the range of 0.60–0.69, acceptable in the range of 0.70–0.79, and excellent if ≥0.80 (51). Determination of the optimal cut point for conversion to a binary categorization of high risk vs. low risk was based on Youden's index for maximum discrimination. Cross-tabulation analyses were performed to assess the statistical significance of exposure–outcome associations (Fisher's exact one-sided *P*) and to calculate classification accuracy statistics, including sensitivity, specificity, and an odds ratio (OR) with its 95% CI for each potential predictor. Interpretation of OR magnitude as small, medium, or large corresponded to values of 1.32, 2.38, and 4.70, respectively (52). Backward stepwise logistic regression analysis was used to identify the best multivariable set of continuous or binary predictors, which included interaction effects between predictors. To avoid overfitting, the prediction model was limited to a 10:1 ratio of criterion-positive cases (i.e., CLEI) to the number of predictors. The intrinsic credibility of the OR associated with the prediction was assessed by comparison to the 95% skepticism limit, which represents both OR magnitude uncertainty and the margin by which it excludes a null effect (53). The analyses were first conducted with data derived from the full mixed-sex cohort. If sex was found to have a significant association with CLEI occurrence (i.e., *P* < 0.05) or to have a significant interaction with another predictive factor, sex-specific analyses of exposure–outcome associations were performed, and independent *t*-tests were used to compare groups. The Shapiro–Wilk test was used to assess data distribution normality, and natural log transformation was used to improve the normality of data that demonstrated a statistically significant (i.e., *P* < 0.05) positive skew.

## Results

**Figure 2:**
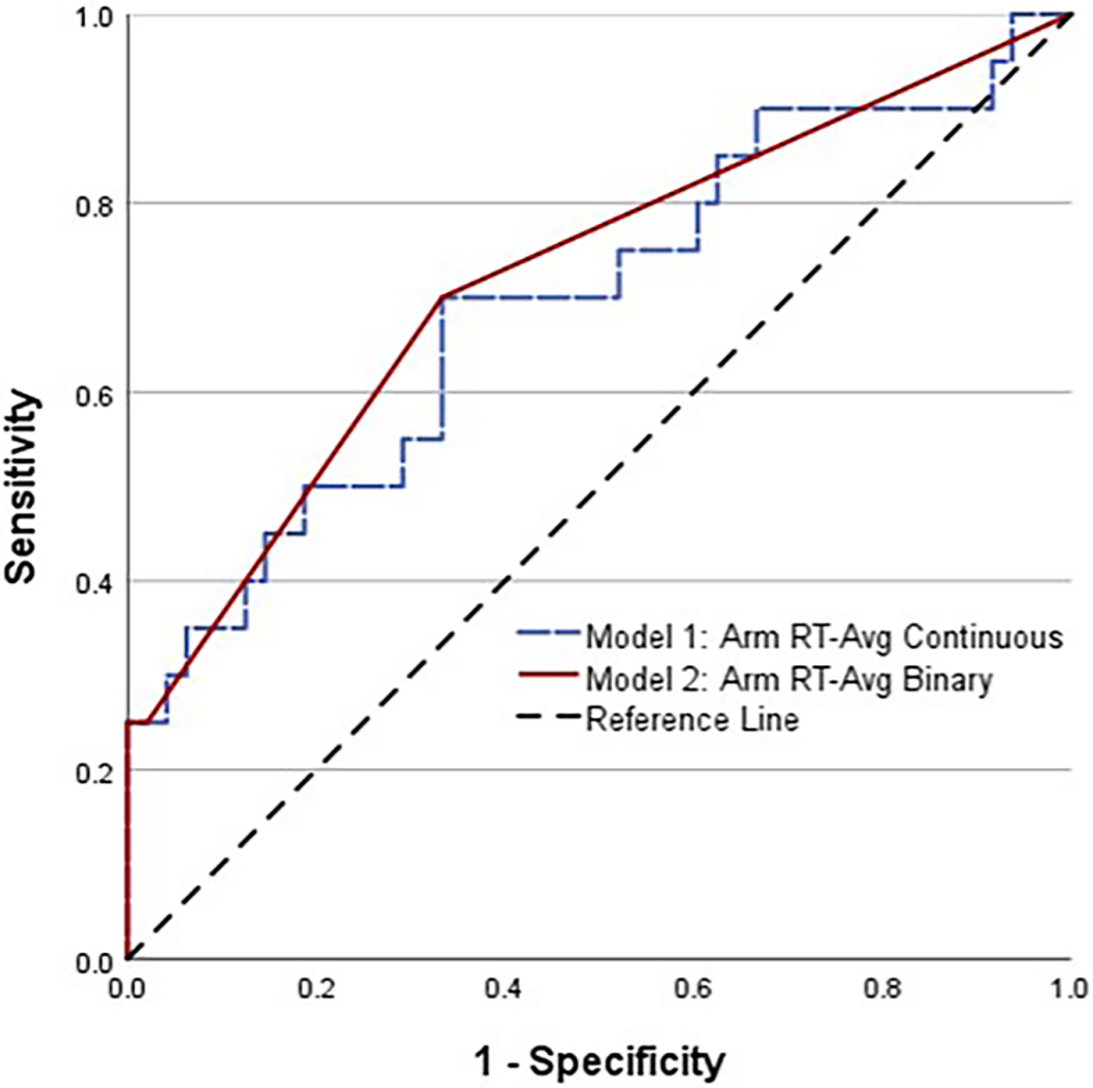
Comparison of receiver operating characteristic (ROC) curves for high school female soccer players with a history of two or more concussions, combined with average arm RT modeled as a continuous variable (dashed line) and as a binary variable (solid line).

A CLEI was sustained by 29% (20/68) of the athletes between the beginning of preseason practice and the end of the season. The body areas affected included the ankle (12), knee (5), hip/groin (2), and low back (1). Based on AUC and OR values, univariable analyses identified a lifetime history of ≥2 concussions (HxC2+), with Arm RT-Avg and Step RT-IIV as the strongest predictors of CLEI (Table 1). The CLEI incidence was 32% (13/41) for females and 26% (7/27) for males, which was not a statistically significant difference (*P *= .408). Backward stepwise logistic regression analysis identified a strong female sex–HxC2+ interaction, with 83% (5/6) of females with HxC2+ sustaining a CLEI compared to 33% (1/3) of males. To avoid overfitting, the prediction model derived from logistic regression analysis was limited to the best two-factor combination, which included female sex–HxC2+ interaction and Arm RT-Avg (Table 2). To evaluate the possibility for model simplification (54), the analysis was repeated with the inclusion of a binary female sex–HxC2+ classification and Arm RT-Avg ≥1.258 s as a second binary predictor (Table 3). The predicted CLEI probabilities derived for each athlete from the two approaches yielded an identical ROC cut point with 0.70 sensitivity, 0.67 specificity, and OR = 4.67; 95% CI, 1.51–14.43 (Figure 2). The OR estimate is deemed large and intrinsically credible (i.e., exceeding the calculated 95% skepticism limit of 3.37).

**Table 1:** The results of univariable receiver operating characteristic (ROC) and cross-tabulation analyses for core or lower extremity injury (CLEI) among male high school football players (*n* = 27) and female high school soccer players (*n* = 41).

Predictor	AUC	Cut point	*P* ^a^	Sensitivity	specificity	Odds ratio	(95% CI)
History of concussion	–	≥2	.016	0.30	0.94	6.43	(1.42, 29.10)
Arm response time—Avg	.690	≥1.258	.004	0.70	0.69	5.13	(1.65, 15.96)
Step response time—IIV	.652	≥0.301	.005	0.75	0.63	5.00	(1.55, 16.09)
Arm RCS—response rime	.618	≤0.69	.060	0.55	0.69	2.69	(0.92, 7.85)
Neck perceptual latency—IIV	.613	≥0.321	.076	0.60	0.63	2.50	(0.86, 7.28)
History of CLEI prior 12 months	–	≥1	.076	0.60	0.63	2.50	(0.86, 7.28)
Neck perceptual latency—Avg	.610	≥0.673	.240	0.65	0.48	1.71	(0.58, 5.03)
GWBI mood-related 0–10 score	.598	≥2	.111	0.55	0.65	2.23	(0.77, 6.44)
Arm perceptual latency—Avg	.593	≥0.811	.044	0.50	0.75	3.00	(1.01, 8.96)
History of concussion	–	≥1	.198	0.50	0.65	1.82	(0.63, 5.25)
Female sex	–	–	.408	0.65	0.42	1.33	(0.45, 3.92)

^a^ Fisher's exact one-sided test.

**Table 2:** Two-factor logistic regression model for core or lower extremity injury (CLEI) including interaction of binary factors (female sex–history of ≥2 concussions) and arm response time—Avg modeled as a continuous variable.

Factor	*B* coefficient	Standard error	Wald *P*	Exp (*B*)	(95% CI)
Female sex–history of ≥2 concussions	2.24	1.20	.076	9.43	(0.90, 98.57)
Arm response time—Avg	2.54	1.43	.061	12.68	(0.77, 210.17)
Constant	−4.36	1.86	.019	0.13	

Model *χ*^2^ (2) = 11.68 (*P *= .003); Hosmer and Lemeshow *χ*^2^ (8) = 7.28 (*P *= .507); Nagelkerke *R*^2^ = .225.

**Table 3:** Two-factor logistic regression model for core or lower extremity injury (CLEI) including interaction of binary factors (female sex–history of ≥2 concussions) and arm response time—Avg modeled as a binary variable (≥1.258 s).

Factor	*B* coefficient	Standard error	Wald *P*	Exp (*B*)	(95% CI)
Female sex–history of ≥2 concussions	2.34	1.18	.047	10.35	(1.50, 71.47)
Arm response time—Avg ≥1.258 s	1.40	0.61	.020	4.07	(1.50, 11.03)
Constant	−1.80	0.46	<.001	0.17	

Model *χ*^2^ (2) = 14.00 (*P *< .001); Hosmer and Lemeshow *χ*^2^ (1) = 0.16; (*P *= .694); Nagelkerke *R*^2^ = .265.

Sex-specific follow-up analyses revealed different relative strengths of univariable predictor associations with CLEI and different cut points for maximum classification accuracy (Tables 4, 5). An effect for GWBI mood-related score was only evident for females. Because data stratification limited the number of criterion-positive cases, sex-specific logistic regression analyses were not performed.

**Table 4:** The results of univariable receiver operating characteristic (ROC) and cross-tabulation analyses for core or lower extremity injury (CLEI) female high school soccer players (*n* = 41).

Predictor	AUC	Cut point	*P* ^a^	Sensitivity	Specificity	Odds ratio	(95% CI)
History of concussion	–	≥2	.008	0.39	0.96	16.88	(1.71, 166.12)
Arm response time—Avg	.695	≥1.385	.029	0.62	0.75	4.80	(1.18, 19.61)
GWBI mood-related 0–10 score	.643	≥7	.045	0.54	0.79	4.28	(1.04, 17.62)
Step response time—IIV	.615	≥0.301	.043	0.77	0.57	4.44	(1.00, 19.75)
Arm RCS—response time	.615	≤0.67	.048	0.62	0.71	4.00	(1.00, 15.99)
Arm perceptual latency—Avg	.573	≥0.881	.066	0.46	0.82	3.94	(0.92, 16.94)

^a^ Fisher's exact one-sided test.

**Table 5:** The results of univariable receiver operating characteristic (ROC) and cross-tabulation analyses for core or lower extremity injury (CLEI) among male high school football players (*n* = 27).

Predictor	AUC	Cut point	*P* ^a^	Sensitivity	Specificity	Odds ratio	(95% CI)
Neck perceptual latency—Avg	.764	≥0.654	.048	0.86	0.60	9.00	(0.90, 89.61)
Arm response time—Avg	.721	≥1.257	.024	0.57	0.90	12.00	(1.48, 97.18)
Step response time—IIV	.721	≥0.282	.048	0.86	0.60	9.00	(0.90, 89.61)
Neck perceptual latency—IIV	.679	≥0.330	.088	0.57	0.80	5.33	(0.83, 34.09)
Arm perceptual latency—Avg	.629	≥0.742	.088	0.57	0.80	5.33	(0.83, 34.09)
Arm RCS—response time	.621	≤0.83	.161	0.86	0.45	4.91	(0.50, 48.62)

^a^ Fisher's exact one-sided test.

Independent *t*-test comparisons of VR performance values for injured vs. uninjured athletes demonstrated a single statistically significant difference for females and multiple statistically significant differences for males (Table 6). Independent *t*-test comparisons of females and males (injured and uninjured combined) identified significant differences for Arm RT-Avg (1.326 ± 0.251 vs. 1.186 ± 0.134; *P *= 0.004), RCS-RT (0.67 ± 0.20 vs. 0.77 ± 0.14; *P *= 0.037), and Arm PL-Avg (0.797 ± 0.168 vs. 0.712 ± 0.116; *P *= 0,025), with males demonstrating better performance for all three VR metrics. Natural log transformation of variables that demonstrated a significant positive distribution skew (Shapiro–Wilk *P* < 0.05) yielded estimated median values (i.e., back-transformation of natural log values) with similar independent *t*-test results for Arm RT-Avg (1.304 vs. 1.179; *P *= 0.006) and Arm PL-Avg (0.781 vs. 0.704; *P *= 0.024).

**Table 6:** Group differences (mean ± SD) between injured [core or lower extremity injury (CLEI) sprain or strain] and uninjured female high school soccer players (13 injured and 28 uninjured) and male high school football players (7 injured and 20 uninjured).

Virtual reality metric	Female soccer players			Male football players		
	Injured	Uninjured	*P* ^a^	Injured	Uninjured	*P* ^a^
Neck perceptual latency—Avg	0.707 ± 0.145	0.748 ± 0.199	.257	0.817 ± 0.353	0.630 ± 0.059	.088
Arm perceptual latency—Avg	0.821 ± 0.187	0.786 ± 0.161	.271	0.760 ± 0.179	0.695 ± 0.084	.101
Step perceptual latency—Avg	0.855 ± 0.213	0.859 ± 0.196	.440	0.895 ± 0.348	0.730 ± 0.091	.**029**
Neck perceptual latency—IIV	0.456 ± 0.418	0.421 ± 0.300	.380	0.881 ± 1.286	0.270 ± 0.094	.087
Arm perceptual latency—IIV	0.467 ± 0.574	0.319 ± 0.240	.124	0.541 ± 0.710	0.244 ± 0.109	.054
Step perceptual latency—IIV	0.534 ± 0.761	0.412 ± 0.348	.240	0.810 ± 1.327	0.324 ± 0.190	.111
Neck response time—Avg	1.134 ± 0.194	1.120 ± 0.225	.405	1.102 ± 0.182	0.934 ± 0.103	.**003**
Arm response time—Avg	1.451 ± 0.282	1.269 ± 0.218	.**015**	1.261 ± 0.156	1.160 ± 0.118	.**042**
Step response time—Avg	1.459 ± 0.219	1.379 ± 0.246	.137	1.386 ± 0.244	1.207 ± 0.112	.**007**
Neck response time—IIV	0.416 ± 0.206	0.390 ± 0.230	.274	0.531 ± 0.457	0.293 ± 0.112	.**018**
Arm response time—IIV	0.494 ± 0.579	0.352 ± 0.260	.140	0.385 ± 0.205	0.285 ± 0.075	.064
Step response time—IIV	0.449 ± 0.404	0.388 ± 0.316	.223	0.560 ± 0.595	0.293 ± 0.085	.**032**
RCS—arm perceptual latency	1.06 ± 0.41	1.09 ± 0.36	.406	1.24 ± 0.31	1.24 ± 0.30	.498
RCS—arm response time	0.62 ± 0.20	0.70 ± 0.19	.100	0.73 ± 0.14	0.78 ± 0.14	.210

^a^ One-sided *t*-test (*P*-value for normally distributed data or natural log transformation of significantly skewed data).

Avg: 40-trial average IIV, 40-trial intraindividual variability; RCS, rate correct score.

Bold values represent injured vs.uninjured differences that are statistically significant (*P* <0.05).

**Figure 3:**
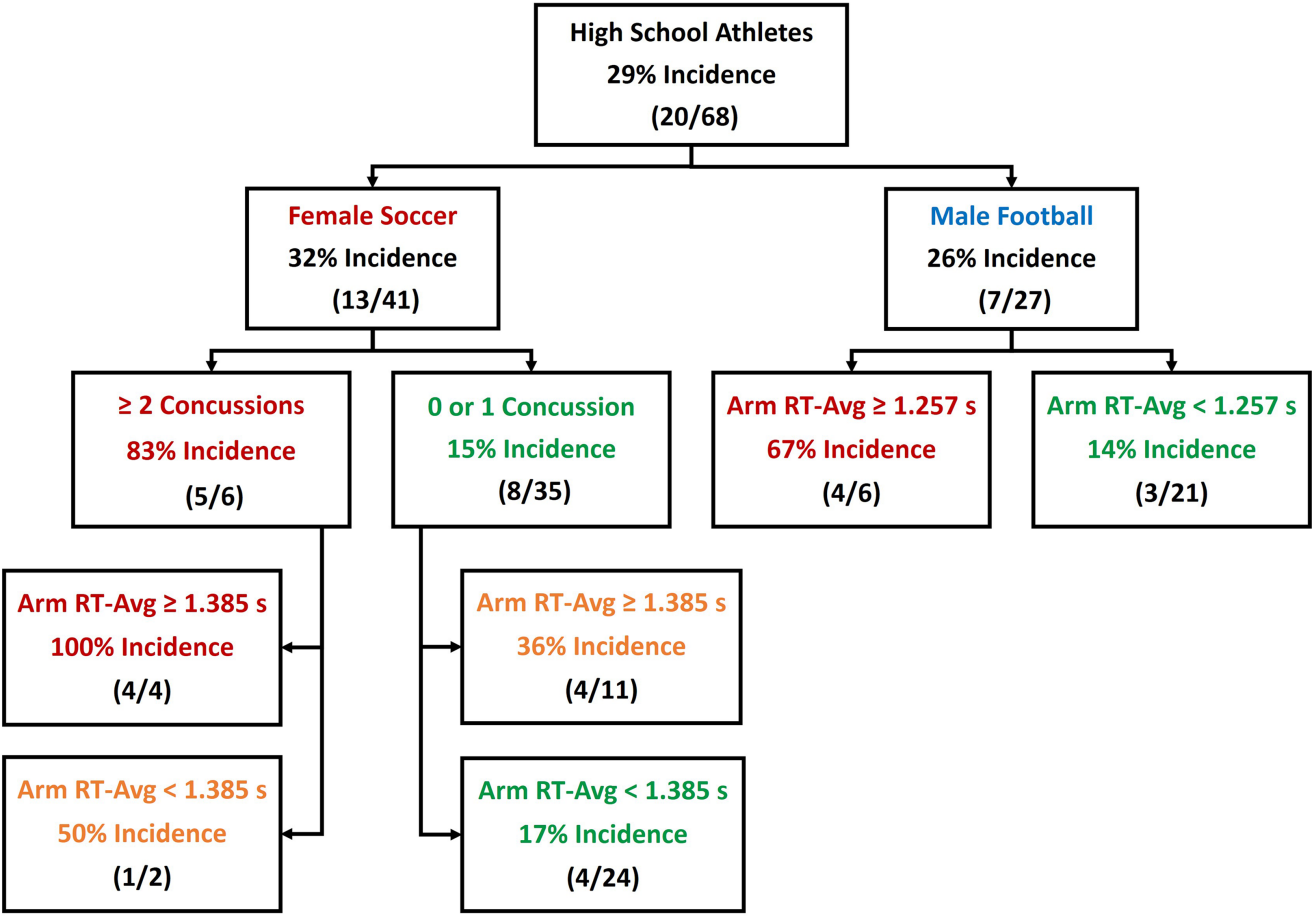
Classification tree depicting accuracy derived from binary predictors of core or lower extremity injury (CLEI) occurrence among female and male high school athletes.

Despite an insignificant univariable association between sex and CLEI occurrence, there was a significant sex–concussion history interaction, and Arm RT-Avg values were significantly different for males and females. A classification flowchart is provided to summarize sex-specific differences in the prediction of CLEI incidence (Figure 3).

## Discussion

Our results are consistent with a previously reported finding of increased odds for lower extremity injury among college athletes with HxC2+ (2) and a finding of greater odds for a history of knee or ankle injury among female college athletes with a history of multiple concussions (4). Furthermore, young adult females with HxC2+ have been reported to exhibit a persisting impairment in visual processing and motor control (12). Because vision is the primary contributor to motor control during sport participation (55), a prolonged motor response to visual stimuli (i.e., slow RT-Avg) may translate to a reduced ability to avoid collisions or prepare to resist unavoidable impending impacts in a competitive sport environment (5, 16, 23, 56). Slow Arm RT-Avg was a strong predictor of CLEI among both male and female athletes. Data stratification by sex demonstrated that males were significantly faster than females (1.186 ± 0.134 s vs. 1.326 ± 0.251 s), and sex-specific cut points demonstrated maximum risk classification accuracy (males ≥1.257 and females ≥1.385 s).

Compared to male athletes, female athletes of all ages have been reported to be more susceptible to concussion and to experience more prolonged post-concussion symptoms of greater severity (57). A recent prospective study of risk for concussion occurrence among college athletes identified a history of at least one concussion as the strongest predictor (58). A logistic regression model that also included impulse control and post-concussion symptom severity was reported to attenuate the effect of female sex on concussion occurrence (58). College students with HxC2+ have reported an incremental increase in the severity of symptoms with each additional concussion, with the largest effect on emotional symptoms following the second concussion (8). The overall results of these studies align well with our findings of univariable associations of HxC2+ and GWBI mood-related scores as strong predictors of CLEI occurrence among female high school athletes (Table 4).

Cognitive, emotional, and behavioral responses to stimuli are determined by functional connectivity patterns within and between brain networks (8, 15). Vulnerability to repeated concussion and increased symptom severity may result from acute microstructural disruption within white matter tracts and a subsequent neuroinflammatory degenerative process (6, 7). An initial concussion can produce an immune system sensitization effect on brain microglia that induces a neuroinflammatory response of increasing intensity with each additional traumatic event (9–11). Many athletes are reluctant to report concussion symptoms and repetitive head impacts that do not produce symptoms that can have similar long-term effects as those of concussion (16, 59), which may result in an unrecognized state of high vulnerability to both subsequent mild traumatic brain injury and musculoskeletal injury. Thus, an important aspect of protecting the health and well-being of each athlete is regular screening for detection of any factor that may increase vulnerability to successive injury events and progressive disability (20, 58).

Co-activations of spatially separated brain areas can be disrupted (i.e., hypoconnectivity) or augmented by compensatory upregulation of neural activity (i.e., hyperconnectivity) (15). Control of goal-directed behavior requires engagement of the central executive network of the brain, which involves salience network suppression of default mode network activity (6, 40). Rapid reconfigurations of network connectivity patterns generate neural signals that are highly variable, which produce consistency in behavioral responses (37, 38). Conversely, reduced default mode network deactivation is believed to result in attention lapses that produce inconsistency in motor output (14). Our finding of an association between elevated Step RT-IIV with CLEI (males ≥0.282 and females ≥0.301) is consistent with evidence for the interpretation of high RT-IIV as a behavioral correlate of neural impairment (20, 27–29, 39).

A key limitation of this study was a sex imbalance that adversely affected the power of stratified follow-up analyses for the detection of associations among the smaller number of male athletes. The lack of exposure tracking for individual participants precluded the calculation of incidence rate (CLEI per 1,000 athlete-exposures), which limited the analyses to comparisons of CLEI incidence. Because concussion history was self-reported, a recall bias may have affected the results. Despite these limitations, we believe our results provide compelling evidence of a need for greater emphasis on clinical testing that is sufficiently sensitive to identify subtle perceptual–motor performance deficiencies that would otherwise remain undetected.

We have demonstrated that VR testing can detect a potentially modifiable contributor to elevated injury susceptibility through documentation of a prospective association between pre-participation perceptual–motor performance and subsequent injury occurrences. A substantial body of evidence has established that concussion effects can persist for months or years beyond clinical recovery and return to sport participation (7, 14, 17, 21). Numerous researchers have presented evidence supporting a need for more stringent criteria to determine post-concussion readiness for return to sport (2, 3, 5, 24, 30) and a need for periodic risk screening of all athletes with a test that imposes sufficient cognitive and motor challenge to reveal subtle deficits (12, 14, 23, 31, 58). Some evidence exists to support the potential for mitigation of injury risk through targeted training (34, 60). More research is clearly needed to document the potential for primary and secondary injury prevention through a properly designed perceptual–motor training program, which is likely to provide the greatest benefit to athletes who exhibit a performance impairment.

## Conclusions

High school female soccer players who have sustained more than one lifetime concussion (HxC2+) appear to be particularly vulnerable to musculoskeletal injury. Further, our prospective cohort study findings support the use of immersive VR to measure the speed, accuracy, and consistency of 40 successive perceptual–motor responses to dynamic visual stimuli. Because the efficiency of perceptual–motor processes may be modifiable, VR training should be investigated as a potentially beneficial approach to the reduction of sport-related injury risk.

## Data Availability

The raw data supporting the conclusions of this article will be made available by the authors, without undue reservation.
